# Changes in bioavailability of sour cherry (*Prunus cerasus* L.) phenolics and anthocyanins when consumed with dairy food matrices

**DOI:** 10.1007/s13197-019-03888-2

**Published:** 2019-07-10

**Authors:** Tugba Oksuz, Zeynep Tacer-Caba, Dilara Nilufer-Erdil, Dilek Boyacioglu

**Affiliations:** 1 Faculty of Chemical and Metallurgical Engineering, Department of Food Engineering, Istanbul Technical University, 34469 Istanbul, Turkey; 2 Department of Food and Nutrition, University of Helsinki, P.O. Box 66, 00014 Helsinki, Finland

**Keywords:** Sour cherry, Food matrix, Bioavailability, Antioxidant, Phenolics, In vitro digestion

## Abstract

In this study, it is aimed to understand the changes in sour cherry phytochemicals when their co-digestions are simulated in dairy model systems comprising skim milk, non-fat-yoghurt, probiotic yoghurt or cream. These co-digestions were analyzed for their total phenolic and anthocyanin contents, total antioxidant activity (TAA) in addition to phenolic and anthocyanin profiles, individually. Sour cherry phenolics were stable during gastric conditions (120%); 54% lost in pancreatic digestion and being available (59%) in serum available fraction (IN). Anthocyanins were lost both in gastric (30%) and pancreatic digestions (16%), being only little available (0.6%) in IN. Soymilk had inhibitory effects on TAA. Dairy food matrix components evaluated were found to have distinct effects on the measured bioavailability of individual sour cherry phenolics. This study might aid both consumers and industry on selecting the food matrices to aiding increase in bioavailability.

## Introduction

Sour cherry (*Prunus cerasus* L.), from *Rosaceae* family, is one of the most significant fruits for the Turkish juice production industry. It is reported to have a high antioxidant potential and phenolic content (Toydemir et al. 2013; Wojdyło et al. 2014). Catechin, epicatechin, quercetin 3-glucoside, quercetin 3-rutinoside, and kaempferol 3-rutinoside may be listed as the major phenolic compounds in sour cherry (Toydemir et al. 2013). They also contain anthocyanins as cyanidin derivatives: cyanidin-3-glucosylrutinoside, cyanidin-3-rutinoside, cyanidin-3-glucoside, cyanidin-3-sophoroside and peonidin-3-glucoside (Wojdyło et al. 2014; Chaovanalikit and Wrolstad 2008; Kong et al. 2003). According to some in vivo studies, cyanidins were reported to be effective in inhibition of cecal tumors and prevention of cell proliferation and tumor growth (Chen et al. 2005; Kang et al. 2003).

However, beneficial effects of anthocyanins were elucidated only when they were consumed in high amounts from 50 to 320 mg/day in the previous intervention studies (Guo and Ling 2015). Therefore, the bioavailability has a key role while evaluating health effects of phenolic compounds and anthocyanins. The extent of absorption and transportation of nutrients to body tissues, might be accepted as the basic definition for bioavailability. There are many factors influencing the bioavailability of antioxidants such as food biological characteristics, components and genotypes; also the gastric conditions (pH, redox potential, presence of any illnesses etc.), food matrix, intestinal absorption, in addition to nutritional and physiological condition and lifestyle (Ercan and El 2010). Therefore, the food matrix, being either able to enhance or prevent the solubilization of anthocyanins during digestion with its structure and composition (Pineda-Vadillo et al. 2017), needs further research.

Dairy foods are one the most commonly consumed products around the world and among all ages. For example, for US consumers, consumption of total dairy products (fluid milk, butter, yoghurt, cheese with evaporated and condensed milk, frozen dairy and dried milk products) has been reported to be as much as around 291.6 kg/per person in 2017 (USDA ERS 2018). Moreover, the market for plant-based products such as soy or almond milk is also increasing. Soymilk is a significant alternative to milk and indicate significant commodity as an alternative to dairy foods.

Previously, some studies worked on how the food matrix affected the bioavailability by combining raspberry was with bread, breakfast, cereal, ice cream and cooked minced beef was studied (McDougall et al. 2005). Studies revealed that total phenolic content of raspberry was found to decrease when it is co-digested with ice cream and breakfast cereals, while no negative effect was evident when consumed with bread or minced meat. Changes in pelargonidin-3-*O*-glucoside bioavailability in strawberry was studied when consumed with and without cream by an in vivo method sampling from plasma and urine. Difference detected between cream-strawberry and single strawberry digestions was not statistically significant (Mullen et al. 2008). Another study evaluated the food matrix affecting the bioaccesibility of pomegranate phenolics and anthocyanins (Sengul et al. 2014). Researchers revealed that bioavailability of pomegranate anthocyanins in serum fraction increased when it is co-digested with carbohydrates and fatty acids however no effect was evident in the case of proteins. On the other hand, for TPC in serum fraction, carbohydrate co-digestions had negative effects (twofold decreases) (Sengul et al. 2014). Effect of presenting anthocyanin fortified dairy matrix as a tool to increase the anthocyanin bioavailability has been studied (Pineda-Vadillo et al. 2017).

However, there is only limited research on the relations between the sour cherry phenolic compounds and their in vitro bioavailability when they are consumed with dairy foods. Therefore, the objective of this study is to evaluate the effects of consuming some dairy foods specifically with sour cherry as a matrix effect on bioavailability of its phenolics and anthocyanins. This research is the first one to elucidate the matrix effect of dairy foods on sour cherry fruit’s health promoting effects.

## Materials and methods

### Materials

Sour cherry samples were purchased from a local grocery market, in Istanbul, Turkey in 2014, were milled under liquid nitrogen using a grinder (IKA, AII Germany). Ground samples were stored at − 80 °C until analysis. Skim milk (0.1% fat, 3.1% protein, 5% carbohydrates), non-fat yoghurt (0.15% fat, 4.7% protein, 7.4% carbohydrates), probiotic yoghurt (2% fat, 3.5% protein, 14% carbohydrates) were supplied from a supermarket. In addition, soymilk with 1.8% fat, 3% protein and 2.5% carbohydrates and cream having 35% fat, 2.3% protein, 3.5% carbohydrates were utilized. All the samples were stored under appropriate conditions until the analyses.

### Chemicals and ingredients

Methanol (≥ 99.9%), formic acid (≥ 98%), sodium carbonate (Na_2_CO_3_), sodium hydroxide (NaOH), hydrochloride acid (37%), sodium acetate trihydrate (CH_3_COONa·3H_2_O) and trifluoroacetic acid (99%) (Merck, Darmstadt Germany); Gallic acid (≥ 98%), Folin–Ciocalteu phenol reagent, ethanol (≥ 99.8%), pepsin, pancreatin, bile salts, acetonitrile (99.8%) and sodium bicarbonate (Sigma-Aldrich Steinheim Germany), and potassium chloride (KCl) obtained from Riedel–de Haen (Hannover, Germany) were used. Kuromanin chloride (cyanidin 3-*O*-glucoside, ≥ 96%) were purchased from Extrasynthese (Genay, France) and chlorogenic acid (≥ 98%) from Fluka Chemie (Buchs, Switzerland); quercetin-3-β-d-glucoside (≥ 98%) and callistephin chloride (pelargonidin 3-*O*-glucoside) were purchased from Sigma-Aldrich (Steinheim, Germany) as HPLC standards.

### Extraction of phenolics

Polyphenols were extracted for determining the initial values and determining differences occurred on phenolics, anthocyanins and antioxidant activities between samples before and after in vitro gastrointestinal digestion (in duplicates) (Bino et al. 2005). According to their modified method, five ml of 75% methanol: water solution involving 0.1% formic acid was added to each tube with ground sample (1 g) that weighed under liquid nitrogen and vortexed for 1 min. Then sonication was applied for 15 min (Ultrasonic Cleaner-VWR, USA) followed by centrifuging (Universal 32, Hettich GmbH Co, Germany) at 4000 rpm under 4 °C for 10 min. Extraction procedure was repeated with fresh solvent until 20 ml of extract is collected. Extracts were stored at − 20 °C until the analysis (Bino et al. 2005).

### Model systems prepared for co-digestions

Skim milk, non-fat yoghurt, probiotic yoghurt, soymilk, and dairy cream were added directly into model systems without any pre-treatment.

(1) For solid foods (non-fat yoghurt, probiotic yoghurt, cream); 5 g of ground sour cherry and 5 g of sample were added in a glass beaker with 20 ml of water, and they were mixed. (2) For liquid foods (skim milk and soymilk); 5 ml of test material, 5 g of ground sour cherry and 15 ml of water were added and mixed thoroughly (Sengul et al. 2014).

### Application of in vitro gastrointestinal digestion (GID)

In vitro GID procedure (McDougall et al. 2005), had two steps: gastric digestion and pancreatic digestion. Pepsin-HCl was applied for mimicking gastric conditions and then bile salts and pancreatin were added as the second step. Simulation of digestion and absorption is performed by using enzymes and dialysis tubing (Sigma-Aldrich; Steinheim, Germany), respectively. 1.5 ml pepsin solution (315 units/ml) was added to both control sample and to the model systems prepared. The pH was adjusted to 1.7 using 5 M HCl. The incubations with enzymes were performed in a shaking water bath (Memmert GmbH, Schwabach, Germany) of 37 °C and at 100 rpm for 2 h. After gastric digestion, 2 ml of solution was taken as the PG fraction (representing the part of food leaving the stomach). 4 ml of pancreatin (4 mg/ml) and 4.5 ml of bile salt mixture (25 mg/ml) were added into the remaining solution. A piece of cellulose dialysis tubing (molecular mass out off, 12 kDa) was filled with 20 ml of NaHCO_3_ (1 M) and tied from the ends. After incubation of mixture for 2 h at 37 °C in a shaking water bath (100 rpm), pancreatic digestion was completed. As the OUT fraction, sampling from the outer solution of tubing was made; representing the non-serum available fraction. Sampling from the solution inside the dialysis tubing was taken as the IN fraction; representing the serum available fraction. All PG, IN and OUT samples were kept at − 20 °C until analysis. After thawing, samples were centrifuged for 20 min at 16,000 rpm, and supernatants were used in analyses.

Values for “% Recovery” of all fractions were calculated using the results obtained for methanolic extracts (control). In addition, LOSS for TPC, TAC, and antioxidant activity results were calculated as the difference of the total amounts of IN and OUT fractions from PG values (LOSS = PG–IN–OUT). All digestions were made in duplicates.

Food models which does not involve sour cherry were used as control digestions for evaluating the changes in phenolics. Data obtained from controls and only sour cherry involving samples were summed up for calculating the “expected” contents. Those expected values were then compared to the “obtained” values for individual foods that are co-digested with sour cherry.

### Total phenolic and total anthocyanin contents

The amount of total phenolic were measured using the Folin–Ciocalteau method (Velioğlu et al. 1998). Results were given as gallic acid equivalents (GAE) per 100 g of edible fruit of triplicate measurements.

By analyzing co-digested samples, the expected and obtained values were compared to each other to understand the effects of the food matrix, as the food matrices tested can contain phenolics and/or other compounds that may react with Folin–Ciocalteau reagent.

Total anthocyanin content was measured by pH differential method (Kar et al. 2011). Since no anthocyanins were detected in the dairy foodstuff used; all anthocyanins were assumed to originate only from sour cherry. The results were reported as cyanidin-3-glucoside (cyn-3-gly)/100 g for the edible fruit and as average of triplicate measurements.

### Total antioxidant activity (TAA) measurements

TAA of the samples were estimated by two different spectrophotometric (Shimadzu; Kyoto, Japan) assays. The first method based on the radical scavenging activity of reagent of 1,1-diphenyl-2-picrylhydrazil (DPPH) (Viuda-Martos et al. 2011). The second test method included the reagent of 2,2-azinobis (3-ethylbenzothiazoline)-6-sulphonic acid (ABTS) (Miller and Rice-Evans 1997). Standard curves were prepared for Trolox (6-hydroxy-2,5,7,8-tetramethylchroman-2-carboxylic acid) and the results were presented as trolox equivalents (TE) per 100 g of edible fruit of triplicate assays.

### Analysis of major phenolics and anthocyanins using high performance liquid chromatography (HPLC)-photodiode array (PDA)

Major phenolic compounds and anthocyanins in in vitro digestion samples and individual food materials/ingredients/components were analyzed using the HPLC-PDA by using external standards (gallic acid, chlorogenic acid, neochlorogenic acid, rutin and kuromanin chloride) according to the method by Bino et al. (2005). Following addition of 50 μl trifluroacetic acid (TFA) to extracts, filtration was applied through a 0.45-μm membrane filter. Filtered extract (1 ml) taken into vials was analyzed in a Waters W2695 HPLC system with PDA (Waters 2996) detector. Stationary phase was the Luna C18 column (150 × 4.60 mm pore size 100 Å, particle size 5 µm—Phenomenex, Torrance, CA, USA), while solvent A (Milli-Q water with 0.1% (v/v) TFA) and solvent B (acetonitrile with 0.1% (v/v) TFA) were used as the mobile phase. A linear gradient: at 0 min, 95% solvent A and 5% solvent B; at 45 min, 65% solvent A and 35% solvent B; at 47 min, 25% solvent A and 75% solvent B; and at 54 min it returns to initial conditions was used with 1 ml/min flow rate. Detections were carried out at different wavelengths of 280, 312, 360, and 512 nm and retention times in addition to characteristic UV spectra were used for identifications. External standard calibration curves were used for quantifications. The results of duplicate analyses were given as mg/100 g edible fruit (Bino et al. 2005).

### Statistical analysis

All results were as mean value ± standard deviation and one-way analysis of variance (ANOVA) was performed to compare the significant differences for the (E) and (O) values in food materials/ingredients/food components in sour cherry added and individual samples (*p* < 0.05). Detailed examinations were achieved by using Duncan’s New Multiple Range Test. For total phenolics data obtained after GI digestion, the differences between (E) and (O) values were statistically analyzed by paired t*-*test for each sample (*p* < 0.05). Statistical Package for the Social Sciences software (Version 20.0, SPSS Inc., Chicago, IL) was used for all statistical analyses.

## Results and discussion

### Changes in total phenolic and anthocyanin contents of sour cherry samples during Gastro intestinal digestion (GID)

Total phenolic contents, total anthocyanin contents and total antioxidant activities of sour cherry extracts were measured as control. Two different solvent systems were used for preparing the sour cherry extracts: (1) Methanol:water solution (75%) with 0.1% formic acid and (2) Water (100%) to represent both analytical conditions and digestion conditions, respectively. In vitro GI digestion was applied directly to sour cherry and TPC, TAC and TAA were measured at each fraction; PG, IN, OUT. As an assumption; TPC, TAC and TAA % values of methanolic sour cherry extract was accepted as 100% (Table 1).

**Table 1:** Total phenolic, total anthocyanin contents and total antioxidant activity results of sour cherry extracts and samples before and after in vitro GI digestion with relative distributions (recovery%) between fractions

	TPC%	TPC (mg GAE/100 g edible fruit)	TAC%	TAC (cyn-3-glu/100 g edible fruit)
Initial methanolic extract	100	287.6 ± 51.2^b^	100	66.6 ± 2.2^a^
Initial aqueous extract	147.6	424.4 ± 10.2^a^	16.5	11.0 ± 4.7^c^
In vitro GI digestion samples				
PG	176.7	508.2 ± 52.3^a^	30.2	20.1 ± 4.6^b^
IN	10.1	29.0 ± 12.4^c^	0.6	0.4 ± 0.1^d^
OUT	86.7	249.3 ± 39.0^b^	13.9	9.3 ± 0.2^c^
LOSS	79.9		15.7	

Total antioxidant activity results				
	TAA%	TAA-DPPH (mg TE/100 g edible fruit)	TAA%	TAA-ABTS (mg TE/100 g edible fruit)
Initial methanolic extract	100	1318.2 ± 76.70^a^	100	74.4 ± 6.7^c^
Initial aqueous extract	35.3	465.2 ± 78.38^b^	75.9	56.5 ± 4.6^c^
In vitro GI digestion samples				
PG	106.1	1398.1 ± 10.59^a^	1065.0	792.4 ± 49.1^a^
IN	6.5	86.0 ± 0.9^c^	93.6	69.7 ± 10.1^c^
OUT	2.8	36.4 ± 8.7^c^	694.3	516.6 ± 37.0^b^
LOSS	96.8		277.0	

Values are means of triplicate measurements ± standard deviations. Different letters in the same column represent significant difference at *p* < 0.05. The terms represent; PG, post gastric fraction leaving the stomach; IN, fraction entering the serum-dialyzable fraction; OUT, fraction remaining in the GI tract-undialyzable fraction. LOSS = PG–IN–OUT Loss in pancreatic digestion

Sour cherry methanolic extract had the total phenolic content of 287.6 ± 51.2 mg GAE/100 g fresh fruit, while total phenolic content in the water extract was 424.4 ± 10.2 mg GAE/100 g fresh fruit. In this study, water extract of sour cherry was also evaluated because water is the solvent in digestion system. Water extract of sour cherries was found as 147.56% of the methanolic extract (100%), when relatively compared. The lowest amount of TPC was observed in IN fraction (29.0 ± 12.4 mg GAE/100 g fresh edible fruit) of sour cherry. On the other hand, PG fraction showed the highest TPC (508.2 ± 52.3 mg GAE/100 g fresh edible fruit) in comparison to methanolic extract. Phenolic content increased after gastric digestion and this increasing effect of gastric acid has also been reported previously (Pérez-Vicente et al. 2002). More than half of the TPC measured at the PG was lost at the end of GID (gastrointestinal digestion) (79.9%). This finding for TPC contents in sour cherry during GID is in agreement with the findings for pomegranate (Sengul et al. 2014). The only difference was that the losses during GID was higher in sour cherry than that of pomegranate (24.9%) (Sengul et al. 2014). GID has been detected to have similar trend at each fraction for sour cherry sample as reported previously (Toydemir et al. 2013).

Amount of total anthocyanins in sour cherry after gastric digestion (PG) was measured as 20.1 ± 4.6 mg cyn-3-glu/100 g fresh edible fruit, while in serum fraction (IN) 0.4 ± 0.1 mg cyn-3-glu/100 g fresh edible fruit and in colon (OUT) 9.30 ± 0.2 mg cyn-3-glu/100 g fresh edible fruit. Methanolic sour cherry extracts showed sixfold higher TAC contents than aqueous extract. Losses during GI digestion were significantly lower for TAC content (15.7%) with respect to TPC contents. TAC recovery values were lower than that for TPC, in parallel with the previous studies (Toydemir et al. 2013; Sengul et al. 2014).

TAA determined by DPPH method, for sour cherry methanolic extract was 1318.2 ± 76.7, while it was 465.2 ± 78.4 mg TE/100 g edible fruit for aqueous extract. Highest TAA was obtained for PG fraction (1398.1 ± 10.6 mg TE/100 g edible fruit) followed by OUT (36.4 ± 8.7 mg TE/100 g edible fruit) and IN (86.0 ± 0.9 mg TE/100 g edible fruit) fractions. The loss in antioxidant activity was considerably significant (96.8%) (*p* < 0.05).

TAA measured by ABTS method for methanolic extract was 74.4 ± 6.7 mg TE/100 g fresh fruit. TAA for PG (792.4 ± 49.1 mg TE/100 g fresh fruit) was close to that of methanolic extract and for IN conditions (69.7 ± 10.1 mg TE/100 g fresh fruit) the measured value was the lowest. Antioxidant activities of sour cherries (34 different cultivars) were studied by ABTS method and the results ranged between 225.3 ± 77.6 and 1576.8 ± 187.6 mg TE/g fresh fruit (Khoo et al. 2012), in consistent with present study. According to the evaluation of TAA of traditional Portugal cherries by oxygen radical absorbance capacity (ORAC) method (in in vitro conditions), the results were in the range of 1651.9 ± 125.6–4430.1 ± 175.2 mg TE/g dry weight (Serra et al. 2010). Antioxidant properties of sour cherry cultivars have also been reported in terms of radical scavenging activity (%) of sour cherry as 96.4% for 0.5 g of fresh edible fruit by DPPH radical and 95.0% by ABTS radical for 0.5 g of fresh edible fruit (Piccolella et al. 2008).

### Changes in major phenolics and anthocyanin compounds in sour cherry during GID

Major phenolics and anthocyanins identified in sour cherry samples before and after GI digestion are shown in Table 2. Chlorogenic acid (85.04 ± 6.00 mg/100 g edible fruit), rutin (24.43 ± 1.34 mg/100 g edible fruit), neochlorogenic acid (17.26 ± 1.67 mg/100 g edible fruit) and gallic acid (8.90 ± 0.83 mg/100 g edible fruit) were the major phenolics detected in methanolic extracts. For each phenolic compounds significant differences were obtained between the extracts and the fractions (*p* < 0.05). However, between IN and OUT fractions no significant difference was seen for these phenolics (*p* > 0.05).

**Table 2:** Changes in major phenolics and anthocyanins of sour cherry extracts and samples before and after in vitro GI digestion

Major phenolics (mg/100 g edible fruit)				
	Gallic acid	Neochlorogenic acid	Chlorogenic acid	Rutin
Methanolic extract	8.90 ± 0.83^a^	17.26 ± 1.67^b^	85.04 ± 6.00^a^	24.43 ± 1.34^a^
Aqueous extract	0.41 ± 0.10^b^	8.47 ± 2.96^c^	2.60 ± 1.48^c^	ND
Bioavailability of in vitro digested sour cherry samples				
PG	0.05 ± 0.01^b^	27.34 ± 0.19^a^	10.05 ± 0.07^b^	22.31 ± 0.15^a^
IN	0.46 ± 0.00^b^	0.10 ± 0.02^d^	0.32 ± 0.17^c^	ND
OUT	0.75 ± 0.04^b^	0.50 ± 0.07^d^	1.00 ± 0.09^c^	ND

Major anthocyanins (mg/100 g edible fruit)		
	Cyanidin-3-O-glucoside	Cyanidin-3-(2G-glucosylrutinoside)
Methanolic extract	15.67 ± 0.57^a^	8.90 ± 0.83^a^
Aqueous extract	0.70 ± 0.24^c^	0.41 ± 0.10^b^
GI in vitro digested sour cherry samples		
PG	6.56 ± 0.11^b^	0.05 ± 0.02^b^
IN	0.02 ± 0.01^c^	0.24 ± 0.02^b^
OUT	ND	0.75 ± 0.04^b^

Values are means of triplicate measurements ± standard deviations. Different letters in the same column represent significant difference at *p* < 0.05. The terms represent; PG, post gastric fraction leaving the stomach; IN, fraction entering the serum-dialyzable fraction; OUT, fraction remaining in the GI tract-undialyzable fraction. ND, not detected

Among phenolic compounds evaluated; the solubility of phenolics in methanolic extract were significantly higher than that in aqueous extracts (*p* < 0.05). For gallic acid, higher bioavailability was observed in serum (0.46 ± 0.00 mg/100 g edible fruit) and colon (0.75 ± 0.04) than in post gastric fraction (PG) (0.05 ± 0.01). In contrast, neochlorogenic acid showed highest content (27.34 ± 0.19 mg/100 g edible fruit) in PG fraction. Similarly, the bioavailability is significantly lower in both OUT and IN fractions in comparison to PG fraction for the other phenolics detected (*p* < 0.05). Neochlorogenic acid has been reported to change in the range of 6.74–27.79 mg/100 g among different species of sour cherry (Kim et al. 2005). Chlorogenic acid result for the methanolic extract was extremely higher (about eight fold) than that in post gastric condition. About 86.8% loss was observed after gastric digestion. As t rutin was not soluble in water, no peak was identified in IN and OUT conditions. The result for rutin in methanolic extract and PG sample was close to each other so acidic condition might be affecting the polarity and increasing the solubility in water. It has been reported that (Jakobek et al. 2009), the major phenolic acids in sour cherry were: chlorogenic acid > rutin > neochlorogenic acid similar to the findings in this present study. Besides, gallic acid was also detected, possibly depending on the different species analyzed.

Two major anthocyanins cyanidin-3-O-glucoside (15.67 ± 0.57 mg/100 g edible fruit) and cyanidin-3-(2G-glucosylrutinoside) (8.90 ± 0.83 mg/100 g edible fruit) were detected. Results showed that anthocyanin has considerably lower solubility in water than in methanolic solution. Anthocyanins were stable at acidic conditions (McDougall et al. 2007), but their degradation was obvious after gastric digestion. Cyanidin-3-(2G-glucosylrutinoside) was much more stable than cyanidin-3-O-glucoside during pancreatic digestion. Previous research revealed that the low levels of anthocyanins recovery during pancreatic digestion and increase in the amounts of phenolic compounds might be related with the conversion of anthocyanins into phenolic compounds (McDougall et al. 2007). Amounts of cyanidin-3-O-glucoside in processed (frozen and dried respectively) Hungarian sour cherries have been reported as 7 mg/100 g and 133 mg/100 g; and for cyanidin-3-(2G-glucosylrutinoside) as 3.36 mg/100 g and 125.87 mg/100 g (Kirakosyan et al. 2009). In another study, amount of cyanidin-3-O-glucoside has been measured between 88.95 and 227.66 mg/100 g and cyanidin-3-(2G-glucosylrutinoside) was between 1.43 and 12.06 mg/100 g in different Hungarian sour cherry varieties of (Kim et al. 2005). Turkish sour cherry, in the present study, was found to possess lower cyanidin-3-O-glucoside content in comparison to Hungarian sour cherries, although thier cyanidin-3-(2G-glucosylrutinoside) level was similar.

### Food matrix effect on sour cherry bioavailability

Variations in total phenolic content (TPC), total anthocyanin contents (TAC) and antioxidant activities (TAA) before and after consuming sour cherry with dairy food samples (skim milk, non-fat yoghurt, probiotic yoghurt, cream and soymilk—as a dairy alternative) were measured.

### Effect on TPC

According to the comparison between the expected and obtained results (depicted in Table 3), in PG fractions generally inhibitory effects were evident except for soymilk co-digestion, while for IN and OUT fractions mostly no change was observed (therefore, the sour cherry phenolics have not significantly affected (*p* < 0.05) the obtained phenolic acid contents. Only exceptions were for milk and soymilk in OUT fraction (promoting effects).

**Table 3:** Total phenolic contents (mg GAE/100 g edible fruit) measured by Folin–Ciocalteau method after co-digesting sour cherry (SC) with other foods and components

	PG		IN		OUT	
	Effect		Effect		Effect	
SC control	508.2 ± 52.3^b^		28.99 ± 12.4^a^		249.28 ± 39.01^c^	
SC + Skim Milk						
O	433.3 ± 36.1^bc^	I	46.4 ± 6.5^a^	U	436.6 ± 51.0^b^	P
E	568.0 ± 53.4		54.8 ± 13.5		302.6 ± 36.7	
SC + Yoghurt						
O	493.1 ± 34.3^b^	U	56.1 ± 4.0^a^	U	326.1 ± 7.8^c^	U
E	572.9 ± 52.2		57.9 ± 8.3		310.8 ± 39.9	
SC + Probiotic Yoghurt						
O	419.6 ± 91.7^bc^	I	57.5 ± 23.6^a^	U	315.8 ± 42.5^c^	U
E	596.8 ± 41.0		49.8 ± 18.3		304.8 ± 37.7	
SC + Soy Milk						
O	1546.6 ± 47.3^a^	P	36.3 ± 11.0^a^	I	528.9 ± 16.3^a^	P
E	560.2 ± 48.5		57.3 ± 12.0		297.2 ± 37.9	
SC + Cream						
O	309.4 ± 30.8^c^	I	34.6 ± 11.3^a^	I	309.4 ± 30.8^c^	I
E	552.4 ± 57.3		44.5 ± 10.7		316.2 ± 33.1	

Values are means of triplicate measurements ± standard deviations: the terms represent; PG, post gastric fraction leaving the stomach; IN, fraction entering the serum-dialyzable fraction; OUT, fraction remaining in the GI tract-undialyzable fraction. Paired t-test was applied between expected and obtained values. When the difference was insignificant: U—unaffected, when the difference was significant if O < E: I—inhibiting effect, if O > E: P—promoting effect

When all co-digestions are evaluated, yoghurt food matrix seemed to have no effect on phenolic bioavailability at all fractions (*p* > 0.05) while cream has inhibitory effects (*p* < 0.05) at all fractions. Similar effects were observed with other fermented products in recent literature. No differences have been detected among total phenolic compounds in detected during different stages of digestion, in the kefir with aronia (*Aronia melanocarpa*, a polyphenol-rich berry that is native to North America) (Du and Myracle 2018). The unique effect of cream might be related with its higher fat content.

Chocolate consumption with milk has been linked to a decrease in flavonoid bioavailability due to dual bonds formed between chocolate flavonoids and milk proteins (Serafini et al. 2009). In another study, effect of milk on dietary polyphenols in tea was studied by an in vivo method and decreasing effect of semi-skimmed milk on the bioavailability of tea polyphenols was reported (Langley-Evans 2000a).

### Effect on TAC

**Fig. 1:**
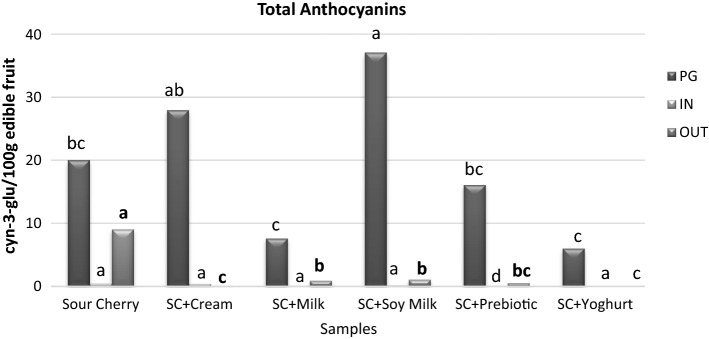
Changes in total anthocyanin contents (mg cyn-3-glu/100 g fresh weight) in each fraction obtained from in vitro GI digestion after simulating consumption of sour cherry with other foods. SC, sour cherry; PG, fraction leaving the stomach; IN, fraction absorbed in serum; OUT, fraction not absorbed in serum. Different letters for foodstuffs in the same series represent significant difference at *p* < 0.05

None of the co-digestions increased bioavailability of anthocyanins in IN fraction (Fig. 1). In contrast, yoghurt and milk as food matrices had decreasing effects on the anthocyanin bioavailability. This effect might be related with the interaction and great affinity between milk proteins and anthocyanins (Trigueros et al. 2014). Soymilk increased the bioavailability of anthocyanin at the PG fraction. Similar effects of soymilk on the bioavailability of phenolic compounds was mentioned in previous section. Difference was not statistically significant (*p* > 0.05) in IN fraction, while SC + Yoghurt and SC + Milk showed the lowest values. All samples of cream, milk, soymilk, yoghurt and probiotic yoghurt decreased the bioavailability of anthocyanins, in the OUT fraction. According to a study on anthocyanin bioavailability during strawberry co-digestion with cream in vivo, pelargonidin-3-glucoside content in serum did not change during 0–24 h whereas it dropped between 0 and 2 h in single material strawberry. Increase in pelargonidin-3-glucoside content was evident after 5–8 h following the consumption of cream and this was related with cream functioning as an increasing factor for the time necessary for strawberry digestion (Mullen et al. 2008).

### Effects on TAA

Total antioxidant activity (TAA) results obtained by DPPH method were given in Table 4a. When expected and obtained values were compared; soymilk with sour cherry were found to have inhibitory effect in all PG, IN and OUT fractions. Nearly all combinations had inhibitory effects in IN conditions, except for cream and probiotic yoghurt which had no effects.

**Table 4:** Changes in (a) antioxidant activity by DPPH method, (b) total antioxidant activity by ABTS method for sour cherry (SC) after co-digestion with other foods

	PG		IN		OUT	
	(mg TE/100 g fresh fruit)					
	Effect		Effect		Effect	
*(a)*						
SC control	1398.1 ± 10.6^a^		86.0 ± 0.9^ab^		36.4 ± 8.7^d^	
SC + Milk						
O	1361.1 ± 2.0^a^	I	62.5 ± 11.5^bc^	I	134.1 ± 50.2^c^	P
E	1529.0 ± 3.7		87.1 ± 2.4		45.1 ± 13.2	
SC + Yoghurt						
O	1117.7 ± 11.7^b^	I	56.9 ± 17.7^c^	I	381.7 ± 33.4^a^	U
E	1627.4 ± 67.1		95.7 ± 11.3		38.0 ± 11.0	
SC + Probiotic Yoghurt						
O	1292.7 ± 71.8^ab^	I	64.6 ± 17.1^bc^	U	217.0 ± 13.2^b^	I
E	1775.5 ± 3.3		90.6 ± 7.3		145.7 ± 14^b^	
SC + Soy Milk						
O	661.5 ± 5.9^c^	I	4.4 ± 0.6^d^	I	33.9 ± 2.1^d^	I
E	1499.3 ± 37.7		187.2 ± 27.2		69.6 ± 8.7	
SC + Cream						
O	1340.5 ± 9.1^a^	I	100.2 ± 11.5^a^	U	175.0 ± 6.2^bc^	U
E	1422.7 ± 18.1		92.7 ± 2.1		136.2 ± 40.1	
*(b)*						
SC control	792.4 ± 49.1^ab^		69.7 ± 10.1^d^		516.6 ± 37.0^c^	
SC + Milk						
O	995.4 ± 12.7^ab^	U	102.6 ± 1.3^bc^	I	582.9 ± 47.7^abc^	I
E	983.34 ± 49.22		195.5 ± 13.8		710.8 ± 34.7	
SC + Yoghurt						
O	986.5 ± 250.1^ab^	I	102.0 ± 4.1^bc^	I	641.7 ± 20.3^a^	I
E	2120.1 ± 34.3		232.9 ± 5.3		1494.7 ± 270.1	
SC + Probiotic						
O	833.9 ± 9.6^ab^	I	139.5 ± 22.1^a^	U	525.3 ± 6.5^bc^	I
E	1316.2 ± 67.4		182.2 ± 28.5		1106.9 ± 57.6	
SC + Soy Milk						
O	698.1 ± 172.4^b^	I	78.3 ± 0.4 ^cd^	I	334.8 ± 19.5^d^	I
E	986.3 ± 55.6		187.6 ± 3.3		693.9 ± 40.4	
SC + Cream						
O	1050.6 ± 28.6^a^	I	113.29 ± 2.03^b^	I	589.1 ± 6.1^ab^	I
E	1216.0 ± 39.1		156.3 ± 8.0		1315.3 ± 44.3	

Values are means of triplicate measurements ± standard deviations: the terms represent; P, pomegranate. PG, post gastric fraction leaving the stomach; IN, fraction entering the serum-dialyzable fraction; OUT, fraction remaining in the GI tract-undialyzable fraction. Paired t-test was applied between expected and obtained values. When the difference was insignificant: U—unaffected, when the difference was significant if O < E: I—inhibiting effect, if O > E: P—promoting effect

Researchers previously worked on the results of milk additions on the antioxidant activity of black tea (English milky tea preparation procedure) by FRAP method (Langley-Evans 2000b). The results revealed that all types of milk used in the study decreasing effects on the antioxidant activity of black tea samples. They explained this effect by antioxidant complex mechanisms as milk proteins interacted with tea flavonoids. According to them, the main effect was not directly related to cow milk’s proteins but also to the milk fat. Catechin, being the fat soluble component, in addition to other reactive oxygen species were reported to interact with fat and negative effect on antioxidant activity was related to this interaction (Langley-Evans 2000b).

On the other hand, for OUT fraction, milk induced positive effects on TAA levels. Previously, availability of fat or fatty acids seemed to promote TAA levels in serum fraction (Pineda-Vadillo et al. 2017).

Most of the co-digestions had negative effects on TAA levels. For IN fraction, probiotic yoghurt and cream were the only ones exerting no effect (*p* > 0.05), while all other co-digestions decreased the TAA levels (*p* < 0.05). However, TAA levels in OUT fraction was less affected from co-digestions; inhibitory effects were observed for probiotic yoghurt, soymilk (*p* < 0.05). While, yoghurt and dairy cream showed no effect (*p* > 0.05).

In respect of ABTS results presented in Table 4b, in comparison with sour cherry as the control, the lowest TAA value was observed for soymilk combinations with sour cherry in PG.

Almost of the combinations had inhibitory effects on TAA levels in PG (*p* < 0.05), except for milk, which had no effects (*p* > 0.05). For TAA values in IN and OUT fraction similar inhibitory effects were observed (*p* < 0.05), except for probiotic yoghurt, in which its combination with sour cherry which had no effects (*p* > 0.05).

In literature, polyphenols and proteins in addition to their effects on antioxidant activity have been studied using the ABTS radical scavenging method and the proteins have been reported to be masking the real antioxidant activity level; which was in accordance with our findings (Arts et al. 2002).

### Food matrix and food component effect on major individual phenolic compounds and anthocyanins

Major individual phenolic compounds and anthocyanins detected after GID were given in Table 5a, b, respectively. Detected compounds were; gallic acid, neochlorogenic acid, chlorogenic acid, rutin, cyanidin-3-O-glucoside and cyanidin-3-(2G-glucosylrutinoside).

**Table 5:** Food matrix effect on (a) major phenolic acids, (b) major anthocyanins

	Gallic acid	Neochlorogenic acid	Chlorogenic acid	Rutin
*(a)*				
SC control				
PG	0.05 ± 0.01^b^	27.34 ± 0.19^ab^	10.05 ± 0.07^a^	22.31 ± 0.15^a^
IN	0.46 ± 0.00^a^	0.10 ± 0.02^a^	0.32 ± 0.17^ab^	ND
OUT	0.75 ± 0.04^a^	0.50 ± 0.07^ab^	1.00 ± 0.09^a^	ND
SC + Milk				
PG	0.64 ± 0.15^a^	0.58 ± 0.00^c^	ND	ND
IN	0.32 ± 0.02^a^	0.13 ± 0.09^a^	0.17 ± 0.01^b^	ND
OUT	0.56 ± 0.19^a^	0.01 ± 0.00^c^	0.72 ± 0.05^ab^	ND
SC + Non-fat Yoghurt				
PG	0.44 ± 0.07^a^	23.58 ± 2.96^b^	9.31 ± 1.52^a^	14.16 ± 1.13^ab^
IN	0.47 ± 0.19^a^	0.12 ± 0.04^a^	0.28 ± 0.08^ab^	0.14 ± 0.06^a^
OUT	0.5 ± 0.0^a^	0.43 ± 0.00^bc^	0.85 ± 0.14^ab^	1.83 ± 0.90^a^
SC + Probiotic Yoghurt				
PG	0.58 ± 0.23^a^	30.39 ± 1.31^a^	12.37 ± 0.83^a^	18.42 ± 0.67^a^
IN	0.59 ± 0.35^a^	0.20 ± 0.01^a^	0.44 ± 0.01^a^	0.06 ± 0.01^ab^
OUT	0.63 ± 0.07^a^	0.57 ± 0.01^a^	1.20 ± 0.08^a^	0.95 ± 0.34^ab^
SC + Soy Milk				
PG	0.51 ± 0.00^a^	2.51 ± 0.00^c^	11.72 ± 0.00^a^	17.35 ± 0.0^a^
IN	0.45 ± 0.07^a^	0.12 ± 0.06^a^	0.27 ± 0.13^ab^	0.06 ± 0.00^ab^
OUT	1.17 ± 0.90^a^	0.68 ± 0.07^a^	1.45 ± 0.18^a^	0.41 ± 0.15^b^
SC + Cream				
PG	0.53 ± 0.03^a^	25.98 ± 3.90^ab^	10.32 ± 2.54^a^	12.55 ± 2.5^bc^
IN	0.23 ± 0.00^a^	0.17 ± 0.00^a^	0.36 ± 0.00^ab^	ND
OUT	0.65 ± 0.02^a^	0.61 ± 0.02^a^	1.32 ± 0.02^a^	0.68 ± 0.03^b^

	Cyanidin-3-O-glucoside	Cyanidin-3-(2G-glucosylrutinoside)
*(b)*		
SC control		
PG	6.48 ± 0.11^a^	0.05 ± 0.01^b^
IN	ND	0.46 ± 0.02^a^
OUT	ND	0.75 ± 0.04^a^
SC + Milk		
PG	ND	0.64 ± 0.15^af^
IN	ND	0.32 ± 0.02^a^
OUT	ND	0.56 ± 0.19^a^
SC + Non-fat Yoghurt		
PG	5.76 ± 0.09^a^	0.44 ± 0.07^a^
IN	ND	0.47 ± 0.19^a^
OUT	ND	0.46 ± 0.01^a^
SC + Probiotic Yoghurt		
PG	8.27 ± 0.43^a^	0.58 ± 0.23^a^
IN	ND	0.59 ± 0.35^a^
OUT	ND	0.63 ± 0.07^a^
SC + Soy Milk		
PG	7.00 ± 0.00^a^	0.51 ± 0.00^a^
IN	ND	0.45 ± 0.07^a^
OUT	ND	1.17 ± 0.10^a^
SC + Cream		
PG	5.98 ± 2.83^a^	0.53 ± 0.03^a^
IN	ND	0.23 ± 0.00^a^
OUT	ND	0.65 ± 0.02^a^

Each letter is representing the differences in between the samples for the same fraction and for the specific phenolic concern (ND, not detected)

According to the results, each of milk, yoghurt, probiotic yoghurt, soymilk, and cream, depicted increasing effects on sour cherry gallic acid bioavailability in PG fractions when consumed together. No significant differences detected (*p* > 0.05) in other fractions (IN and OUT) of samples.

Neochlorogenic was the most dominant polyphenolic compound in sour cherry. Probiotic yoghurt showed the promoting effect on the bioavailability of neochlorogenic acid in PG fraction. Yoghurt and cream had no effects on bioavailability of neochlorogenic acid in PG. In literature, fermentation has been shown as a possible efficient approach to enhance the bioavailability of some phenolic compounds (Zhao and Shah 2016). Moreover, presence of some microorganisms that are able to break down the complex phenolic compounds and the metabolites has been reported as an increasing factor bioactivity (Wang et al. 2012). All other combinations (milk and soymilk) decreased the bioavailability significantly (*p* < 0.05). Dairy combinations with sour cherry made no effect on neochlorogenic acid bioavailability in IN fraction. Milk was the only dairy food materials that decreased bioavailability significantly (*p* < 0.05) in OUT fraction.

When chlorogenic acid was evaluated, generally it was seemed to be stable in gastric conditions whereas in IN and OUT fractions bioavailability decreased. As mentioned in previously (Lafay et al. 2006), chlorogenic acid had minor hydrolysis in stomach (< 1%) however, 15–32% hydrolysis was evident into caffeic acid in the cecum. These results were in accordance with TPC results. When consumed with milk, in PG fraction (− 46.87%) chlorogenic acid was either lost or decreased which can be explained by the phenolic compound binding ability of proteins (Ozdal et al. 2013). In a previous study, there was no differences related to fermentation has been detected for chlorogenic acid and neochlorogenic acid between nonfermented control and aronia kefir (Du and Myracle 2018). Rutin, being another major flavanol in sour cherry, was only identified in the PG fraction in sour cherry control sample. Combinations with protein-based matrices such as milk, yoghurt, probiotic yoghurt and soymilk, decreased bioavailability of rutin.

Two major anthocyanins of cyanidin-3-O-glucoside and cyanidin-3-(2G-glucosylrutinoside) were detected in sour cherry: (Table 5b). According to results for cyanidin-3-O-significantly higher values in PG followed by milk, had inhibitory effects on this compound in PG fraction. For IN and OUT fractions, the compound was detected at very low levels as it is known that anthocyanins have low bioavailability in those fractions. Cyanidin-3-O-glucoside was even lost at all fractions for milk co-digestions with sour cherry.

When the cyanidin-3-(2G-glucosylrutinoside) was evaluated, it was seen that generally this compound was much more retained with respect to cyanidin-3-O-glucoside.

## Conclusion

The findings of this study revealed that the in vitro bioavailability of sour cherry phenolics in dairy food matrix was highly stable, however the anthocyanins showed relatively lower stability, particularly in IN fraction. Effects of different dairy matrices highly depend on the individual phenolic and anthocyanin components of sour cherry. Future studies should also focus on the matrix effect on absorption rate and transformations in the colon for in vivo systems. These results of this study are considered as beneficial for both of consumer and industry perspectives, by directing them to choose the proper dairy food matrices for sustaining higher bioavailability levels.
